# Biodiversity monitoring in bamboo coral assemblages in the North Aegean Sea, eastern Mediterranean Basin

**DOI:** 10.3897/BDJ.13.e135156

**Published:** 2025-08-01

**Authors:** Chrysoula Gubili, Konstantinos Touloumis, Esprit H Saucier, Aristeidis Christidis, Elina Samara, Paraskevi Papadopoulou, Stelios Triantafillidis, Nikolaos Kamidis, Emmanouil Koutrakis

**Affiliations:** 1 Hellenic Agricultural Organisation-DIMITRA, Fisheries Research Institute, Nea Peramos, Kavala, Greece Hellenic Agricultural Organisation-DIMITRA, Fisheries Research Institute Nea Peramos, Kavala Greece; 2 Brigham Young University, Laie, Hawaii, United States of America Brigham Young University Laie, Hawaii United States of America

**Keywords:** octocorals, *Isidella*, deep-sea, species richness, taxonomy, diversity

## Abstract

Deep-sea bamboo coral assemblages are often undescribed in the eastern Mediterranean Sea. Their structural complexity provides essential habitats for many species and promotes high biodiversity that is poorly known. In this study, we examined the effects of coral presence on biodiversity of benthic communities from a scientific trawl survey in the North Aegean Sea carried out in 2018. Genetic analyses did not support the first species identification of the coral as *Isidellaelongata* (Esper, 1788). All searches resulted in matches belonging to *Acanella* specimens, a genus with a disputed taxonomic validity since its initial description. Quantitative data from macrobenthos in coral assemblages revealed the presence of at least 45 species. Additionally, few species of economic importance were exclusively detected in the haul associated with coral assemblages, which also hosted the highest species richness. Our results highlight the importance of such ecosystems as diversity hotspots in the marine environment, which are heavily impacted by human activities and are extremely important to commercial fisheries.

## Introduction

The gradual depletion in shallow coastal resources over the last 20 years has led to a shift in interest towards deep water ecosystems which hold new fish stocks, bioprospecting potential, elements and energy reserves (FAO 2009, Thurber et al. 2014, FAO 2018). Technological advances allowed the exploitation of rich biodiversity grounds by the fishing industry, with destructive effects in deep-sea communities (Pusceddu et al. 2014, Clark et al. 2015, Ferrigno et al. 2017, Pierdomenico et al. 2018). Furthermore, species richness of benthic communities in the deep-sea is not fully described (McClain et al. 2011, Glover et al. 2018, Jakiel et al. 2019) and the real consequences of commercial fisheries are far from understood (Maynou and Cartes 2011, Pusceddu et al. 2014, Thrush et al. 2015). Most deep-water species are fragile and have slow recovery rates, making them extremely sensitive to habitat degradation (McClain and Schlacher 2015, Stuart et al. 2003) and activities such as bottom trawling, whereas deep-sea mining operations could threaten entire seabed assemblages when devoid of effective regulation, environmental impact assessments and mitigation strategies (Vanreusel et al. 2016, Jones et al. 2020, Mitchell et al. 2020).

The impacts of ﬁshing activities in deep-sea coral assemblages and the communities they host are not often the focus of scientific investigations in the eastern Mediterranean Basin (Mytilineou et al. 2014,Gerovasileiou et al. 2019), even though some species are considered Vulnerable Marine Ecosystems (VMEs) indicator taxa and relevant in terms of sustainable management priorities (GFCM 2017, GFCM 2018). The bamboo coral *Isidellaelongata* (Esper, 1788) is the most common species of the Keratoisididae family (Cnidaria: Alcyonacea) in the Mediterranean Sea and despite the species’ early detection (Steindachner 1891), its distribution range is constantly updated (Mastrototaro et al. 2017, Gerovasileiou et al. 2019, Ingrassia et al. 2019, Carbonara et al. 2020, Carbonara et al. 2022, Standaert et al. 2023). Isidids have slow annual growth (Andrews et al. 2009) and a life span reaching up to 400 years (Sherwood et al. 2009); however, no information on life history traits of *I.elongata* across its distribution range is currently available (Carbonara et al. 2020).

*Isidellaelongata* is the only Critically Endangered anthozoan species in the International Union for Conservation of Nature Red List (IUCN; Bo et al. (2015)). Its assemblages create critical habitats for both fish and invertebrates, mainly decapods of high commercial interest (D’Onghia et al. 2010, Maynou and Cartes 2011, Cartes et al. 2013, Mastrototaro et al. 2017, Cartes et al. 2022) and, thus are characterised as “Sensitive Habitat” by the General Fisheries Commission for the Mediterranean Sea (FAO 2009). Therefore, prompt species identification is imperative for the enforcement of protection measures against uncontrolled fishing activities. However, identification confusion between *I.elongata* and *Acanellaarbuscula* (Johnson, 1862) has been reported in the Mediterranean Sea, due to similarities in colony form, sampling location and depth. Such confusion was attributed to the original belief that *I.elongata* is the only species found in the Mediterranean basin (Carpine and Grasshoff 1975).

Despite the documented higher species diversity and abundance in habitats dominated by cold-water corals (Husebø et al. 2002, D’Onghia et al. 2010, Rueda et al. 2019, Carbonara et al. 2020), little is known about the role of bamboo coral forests on species richness and abundance in the eastern Mediterranean Sea. This study aims to report a field observation of an octocoral population in the North Aegean Sea, Greece. The aims of this study were to: i) identify the octocoral species using molecular methods, ii) estimate the density of the colonies and iii) assess the megafauna assemblages associated with the specific coral habitat and compare them to those from sites of similar depth and environmental conditions.

## Materials and methods

### Study area and field sampling

**Figure 1.**
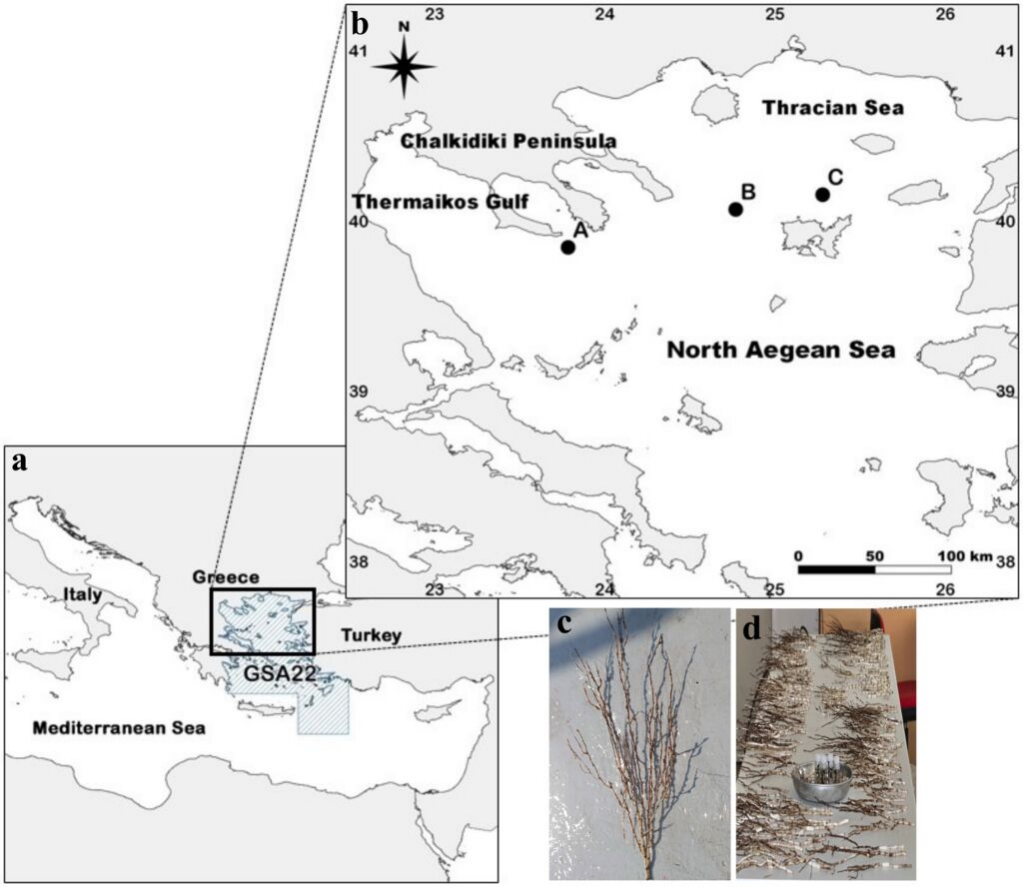
Studied area. **a, b** Approximate sampling locations from the Geographical Sub-Area 22 (GSA 22) and the Mediterranean International Bottom Trawl Survey (MEDITS); **c, d** Samples of bamboo corals collected from the North Aegean Sea during 2018.

Samples were collected at the North Aegean Sea and the Geographical Sub-Area 22 (GSA 22) in 2018 (22^nd^ of June till 11^th^ of July), the most significant fishing ground in the eastern Mediterranean basin (Somarakis et al. 2006), as part of the Mediterranean International Bottom Trawl Survey (MEDITS, Fig. 1, Suppl. material 1). Three hauls were considered for the analysis due to similarities in sampling methodology and proximity of sampling sites, i.e., same depth zone (approximately 580 m), haul duration (1 h) and sampling gear (Sites A, B and C, Fig. 1; for more details see MEDITS Handbook, Anonymous (2017)). Sites B and C are located at the edge of Lemnos basin, while site A is located at the North of Athos basin, in the vicinity of Chalkidiki Peninsula and it hosts aggregations of the bamboo coral *I.elongata* (Fig. 1). All samples (e.g., fishes, crustaceans, cephalopods, Suppl. material 1) were measured (total length, mm) and weighted (total weight, g) and were subsequently identified on board to the lowest possible taxonomic level by scientific observers, when possible. A few individuals were photographed and/or taken to the laboratory to further help in their identification. Few octocoral samples from Site A were immediately stored in -20 °C for further analysis (see Genetic identification). The MEDITS survey follows a standardised protocol as described by Bertrand et al. 2002 and Spedicato et al. 2020 and the MEDITS Handbook (http://www.sibm.it/MEDITS%202011/principaledownload.htm), approved by international authorities (EU).

### Genetic identification

Genomic DNA was extracted from six specimens of bamboo corals from Site A using a NucleoSpin Tissue kit (Macherey-Nagel) according to the manufacturer’s protocol. Fragments of the *igr4* and the *mtMutS* regions were selected as targets for the analysis of each sample, as both markers have been repeatedly used in bamboo barcoding studies due to the high mutation rates (van der Ham et al. 2009). The *igr4* fragment was amplified with the following primers: CytbBam1279f (5′-AGGAGCCAATCCAGTAGAGGAACC-3′) and Nd6Bam1648r (5′-TAYAGGTAAGAAATGCGAGTGATC-3′) from van der Ham et al. (2009). The *mtMutS* sequences were generated using the primers Co3Bam5657f (5′-GCTGCTAGTTGGTATTGGCAT-3′) and MUT3458r (5′-TSGAGCAAAAGCCACTCC-3′) from France (2007). The polymerase chain reaction (PCR) cycling conditions for the ampliﬁcation of both genes included one cycle of 2 min at 94 °C, followed by 35 cycles at 94 °C for 60 s, 56.5 °C for 60 s, 72 °C for 90 s and a ﬁnal extension at 72 °C for 5 min. PCR was conducted in a total volume of 50 μl using 2 μl of DNA template, 10 μl of 5X Promega PCR buffer, 6 μl of MgCl_2_ (25 mM), 1 μl of dNTPs (10 mM, Promega), 1.5 μl of each primer (10 mM), 0.1 μl of Taq Polymerase (GoTaq G2 Flex, Promega) and 27.9 μl molecular grade water. The resulting sequences were checked in Proseq 3.2 (Filatov 2009). Species identification was conducted by similarity-based searches on GenBank (Clark et al. 2015) using the standard nucleotide BLAST (blastn, http://blast.ncbi.nlm.nih.gov/Blast.cgi; Altschul et al. (1990)).

### Data analysis

Despite the disturbance and destruction of seafloor habitat and coral specimens by the otter trawl, an attempt to estimate colony density from Site A was made. Colonies (main trunk) were counted and the total swept area by the trawl net was calculated; therefore, density was estimated in colonies per hectare by considering a range of values (minimum and maximum). Additionally, densities (N individuals km^-2^) and wet biomass (g km^-2^) for each taxon were calculated. Various diversity indices were used to assess biodiversity by quantifying species richness, abundance and abundance evenness (Magurran and Dornelas 2010, Morris et al. 2014); these were the Species richness (S), the Margalef’s richness index (d; Margalef 1958), the Simpson’s dominance (D; Simpson 1949), the Fisher’s a (alpha, Fisher et al. 1943), the Shannon-Wiener’s (H; Shannon and Weaver 1949) and Pielou’s evenness (J; Pielou 1966) for each site. All indices were computed using the “vegan” R package (Oksanen et al. 2014). The statistical significance of the observed differences in fish, crustacean and cephalopod assemblages amongst hauls was evaluated with a non-parametric one-way analysis of similarity (ANOSIM, Clarke and Warwick (2001)), utilising the Bray-Curtis distance measure. Additionally, a similarity percentage analysis (SIMPER; Clarke (2006)) was applied to identify the species responsible for the differences in community composition amongst the sites.

Scientific names assigned to all taxa followed the nomenclature of the World Register of Marine Species, WoRMS (https://www.marinespecies.org).

## Results and discussion

### Genetic identification of octocorals

Partial *igr4* sequences were obtained from all six specimens (amplification and sequencing rate of 100%) varying from 142 to 422 bp (Accession numbers: PV285286-PV285291). Despite the initial identification of the deep-sea octocoral as *I.elongata*, all barcode searches on BLAST resulted in similar multiple top matches. These included *Acanella* spp., *Acanellaeburnea* (Pourtalès, 1868) and *Acanellaarbuscula* with over 99.5% confidence on species assignment. They were followed by species of the family Keratoisididae, including the genera *Orstomisis* and *Lepidisis* (Suppl. material 2). The smallest *igr4* fragment resulted in identical high-top matches on species assignment (100%); however, octocorals from the family Primnoidae also exhibited high confidence on species assignment [e.g., *Plumarellaspinosa* Kinoshita, 1907; *Plumarellaadhaerans* Nutting, 1912; *Narellahawaiiensis* Cairns & Bayer, 2008; *Primnoellachilensis* (Philippi, 1894) etc.]. Similarly, the single *mtMutS* sequence (940 bp; Accession number: PV285292) showed high matches to *Acanella* spp. (99%), followed by *Isidellatentaculum* Etnoyer, 2008 and other species of the family Keratoisididae (Suppl. material 2).

Despite the increase in studies and sample collections of keratoisidins with deep-sea exploration, aspects of their taxonomy and systematics remain poorly understood (van der Ham et al. 2009). Muzik (1978) suggested that the genus *Acanella* should be synonymised with the genus *Isidella*, though it was later disputed by Bayer (1990), based on differences in morphological characteristics. An attempt to corroborate species identification using polyp and sclerite morphology has been attempted in this study (as described in Saucier et al. (2017)); however, it was abandoned as both were damaged during sampling procedures by the trawl nets and the acute increase in temperature (temperature difference ~ 30 ^o^C). Moreover, as the genus *Acanella* has been under taxonomic scrutiny, a thorough taxonomic revision is required to determine whether polyp morphological variability is sufficient for species identification (van der Ham et al. 2009).

### Community composition and diversity variation

A total of 130 colonies were counted, with an additional 28 of unconfirmed identification, estimating a mean density of 15-18 colonies per km^2^. The overall colony density is relatively low compared to other areas with forests in the Mediterranean Sea (Cartes et al. 2013, Bo et al. 2015, Mastrototaro et al. 2017). The colonies were collected from a swept area of approximately 116,571 m^2^ (Site A) and we cannot verify if the colonies were scattered across the swept area, if they were collected from a section of the forest, nor its size. It is, therefore, impossible to know the actual density of the *I.elongata* forest. Additionally, colony sizes could not be fully assessed nor the presence and impact of trawling as in the western Mediterranean Sea (Bo et al. 2015, Pierdomenico et al. 2018,Ingrassia et al. 2019).

At least 64 species from three phyla (Chordata, Arthropoda and Mollusca) were identified amongst the samples collected in the three sites (Suppl. material 1). Of these, 14 species (21.9%) occurred in all hauls and more than half (33 species, 51.6%) occurred only once (19 in Site A, seven in Site B and seven in Site C). One decapod specimen was identified at a genus level, whereas five fish and 12 decapod specimens were identified at family level (Suppl. material 1). The most diverse phylum was Chordata with 39 species, followed by Arthropoda and Mollusca with 15 and nine species, respectively. The most common species in Sites C and B was the roughsnout grenadier [*Trachyrincusscabrus* (Rafinesque, 1810)] which was completely absent from Site A, whilst the hollowsnout grenadier [*Coelorinchuscaelorhincus* (Risso, 1810)] dominated Site A (Suppl. material 1). Additionally, various echinoderms were recorded in all sites [e.g., *Gracilechinusacutus* (Lamarck, 1816), Cidaridae spp.], whilst mäerl was solely detected in Site B. Moreover, the sea pen *Funiculinaquadrangularis* (Pallas, 1766) and 63 *Scyliorhinuscanicula* (Linnaeus, 1758) eggs cases entangled in colonies were found at Site A. The highest density and biomass were recorded by species belonging to the phylum Chordata, followed by the Arthropoda and the Mollusca. Site A also exhibited the lowest biomass amongst the three sites (Table 1). Interestingly, only ten species are of some economic importance for trawl fishery, of which four were exclusively found in Site A (Suppl. material 1). Amongst them, the small-spotted catshark *S.canicula* is not a target species for commercial fisheries, whilst only one specimen of *Nephropsnorvegicus* (Linnaeus, 1758) was caught on Site A (Suppl. material 1). Similarly, other species such as *Anamathiarissoana* (Roux, 1828), have been previously associated with colonies of *I.elongata* (Mastrototaro et al. 2017).

**Figure 2.**
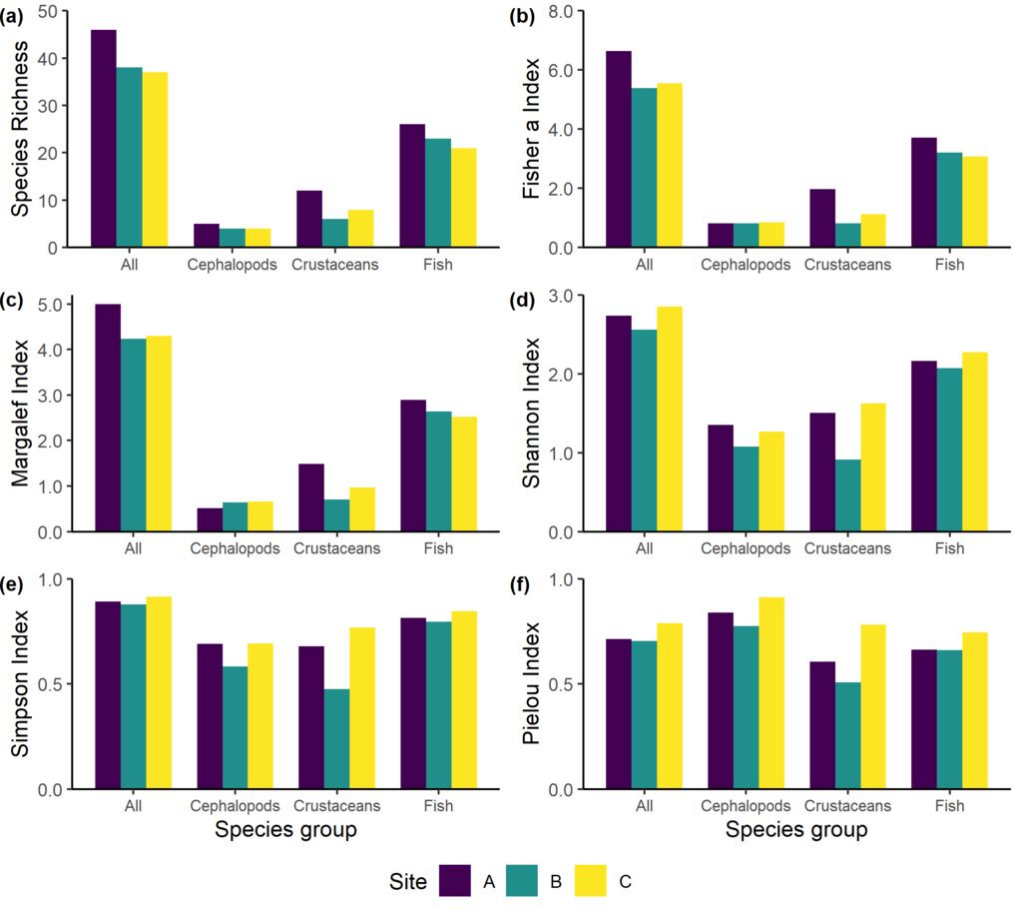
Mean diversity indices from three sampling locations (Sites A, B and C) and three species categories (Cephalopods, Crustaceans and Fish): **a** species richness (number of taxa); **b** Fisher’s a; **c** Margalef’s richness index; **d** Shannon index; **e** Simpson’s dominance index; **f** Pielou’s evenness index.

Species richness ranged from 4 to 26 in the sampling area and the highest values were estimated for all phyla in Site A (Fig. 2) corroborating its importance as a highly important habitat for other species and overall biodiversity (Fabri et al. 2014, Lauria et al. 2017, Mastrototaro et al. 2017). Both Simpson’s and Pielou’s indices were higher for all phyla in Site C. The remaining three indices (Margalef, Fisher a and Shannon) showed mixed results with Sites C and A having the highest values (Fig. 2). Additionally, assemblages differed significantly amongst sites (ANOSIM, R = 1, *p* < 0.003). SIMPER analysis revealed that the roughsnout grenadier (*T.scabrus*) and the roughtip grenadier [*Nezumiasclerorhynchus* (Valenciennes, 1838)] accounted for 16.24% and 11.08% of the similarities, respectively (Suppl. material 3). In total, 14 species accounted for 90% of the observed similarity (Suppl. material 3). Fish assemblage exhibited the highest mean dissimilarity, followed by decapods and cephalopods. Due to the limited sample size (three sites), statistical tests were not conducted. Nonetheless, the estimated diversity indices for the three sites were quite comparable.

Interestingly, the lowest biomass amongst the three sites was exhibited in Site A (Suppl. material 1); however, this was mainly driven by *Trachyrincusscabrus* (Rafinesque, 1810), *Merlucciusmerluccius* (Linnaeus, 1758) and *Centrophorusuyato* (Rafinesque, 1810). All species were absent from Site A and they represented more than half of the total biomass for Sites B and C (Table 1). The first two species have been scarcely reported close to *I.elongata* assemblages (Cartes et al. 2013, Bo et al. 2015, Carbonara et al. 2020), whereas there are no records for *C.uyato* in the area. Additionally, the overall higher species richness, Fisher’s, and Margalef’s values in Site A could be attributed to a more equitable distribution of specimens among the collected species. Among the different taxonomic groups, the largest differences in diversity indices between coral and non-coral habitats were observed in crustaceans. In contrast, the indices for other taxonomic groups remained relatively similar (Fig. 2). However, due to the limited sample size, no statistical tests were performed. High species richness, abundance and biomass of crustaceans have been previously reported in areas with *I.elongata* colonies (Maynou and Cartes 2011, Carbonara et al. 2020, Cartes et al. 2022).

Lower species richness and biomass values (after the removal of three species: *T.scabrus*, *M.merluccius* and *C.uyato*) at Sites B and C could be attributed to higher fishing pressure at both sites compared to Site A. The North Aegean Sea, although superior in terms of productivity, species composition and diversity compared to the South Aegean (Sylaios et al. 2010), is a heavily exploited region that is constantly altered by anthropogenic impact such as fishing activities, while the situation is likely to worsen due to climate change and sea temperature increase (Brander 2007). Many studies denote that most fish stocks in the eastern Mediterranean and specifically in the Aegean Sea (> 60%) are overexploited and/or have collapsed, especially those directly or indirectly targeted by fishing fleets (Vasilakopoulos et al. 2014, Froese et al. 2018).

### Conclusion

Bamboo corals are currently receiving more attention due to their ecological importance and slow recovery. They provide essential habitat for species of commercial interest and enhance biodiversity of benthic ecosystems (Maynou and Cartes 2011, Cartes et al. 2013). Despite the presence of many species in all study sites, the number of taxa observed in Site A is considerably higher than the rest. Moreover, the decreased biomass in coral-associated megafauna was explained by the absence of three species that represented the majority of biomass in Sites B and C. Bottom trawling has a significant effect on megabenthic species and long-term fisheries production. Further studies in the area should focus on data collection in the North Aegean Sea using remote operation vehicles (ROV) and high-resolution bathymetry, temperature, salinity and dissolved oxygen to determine the status of such assemblages. Future management plans are required to protect vulnerable marine ecosystems, particularly in the eastern Mediterranean basin where deep-sea ecosystems are often neglected.

## Supplementary Material

8542420B-88D9-5417-BEFA-756556BF5E1C 10.3897/BDJ.13.e135156.suppl1 Supplementary material 1 List of species and number of individuals registered by sampling site Data type Species list Brief description List of species and number of individuals registered by sampling site. Site A: min 46 species, 772-800 specimens; Site B: min 38 species, 689 specimens; Site C: min 37 species, 552 specimens. * Species of commercial value File: oo_1298675.docx https://binary.pensoft.net/file/1298675 Chrysoula Gubili, Konstantinos Touloumis, Esprit H Soucier, Aristeidis Christidis, Elina Samara, Paraskevi Papadopoulou, Stelios Triantafillidis, Nikolaos Kamidis, Emmanouil Koutrakis

7E7DBD99-9494-52EC-87BE-F1042CDB2C40 10.3897/BDJ.13.e135156.suppl2 Supplementary material 2 BLAST results Data type Genetic data Brief description Details and BLAST results from all specimens used in this study. File: oo_1281728.docx https://binary.pensoft.net/file/1281728 Chrysoula Gubili, Konstantinos Touloumis, Esprit H Soucier, Aristeidis Christidis, Elina Samara, Paraskevi Papadopoulou, Stelios Triantafillidis, Nikolaos Kamidis, Emmanouil Koutrakis

1AB6D5B8-05CA-5AD1-9391-E4DA03234C99 10.3897/BDJ.13.e135156.suppl3 Supplementary material 3 Analysis of similarity percentage (SIMPER) Data type Similarity analysis Brief description Analysis of similarity percentage (SIMPER) for assemblages among Sites A, B and C and of dissimilarity percentage amongst hauls. Contrib. %: contribution in %; Cumulative %: cumulative contribution in %. Species are listed in decreasing order of AD amongst hauls. File: oo_1281730.docx https://binary.pensoft.net/file/1281730 Chrysoula Gubili, Konstantinos Touloumis, Esprit H Soucier, Aristeidis Christidis, Elina Samara, Paraskevi Papadopoulou, Stelios Triantafillidis, Nikolaos Kamidis, Emmanouil Koutrakis

## Figures and Tables

**Table 1.** Density (individuals/km^2^) and biomass (g/km^2^) per species registered by haul. * Species of commercial value.

	**Species**	**Category**	**Site A**		**Site B**		**Site C**	
			**Density**	**Biomass**	**Density**	**Biomass**	**Density**	**Biomass**
1	*Argyropelecushemigymnus* Cocco, 1829	Chordata	25.74	42.89	-	-	-	-
2	*Bellottiaapoda* Giglioli, 1883	Chordata	8.58	17.16	-	-	7.9	23.71
3	*Benthosemaglaciale* (Reinhardt, 1837)	Chordata	-	-	9.04	9.04	55.32	94.83
4	*Centrolophusniger* (Gmelin, 1789)	Chordata	17.16	42034.47	-	-	-	-
5	*Centrophorusuyato* (Rafinesque, 1810)	Chordata	-	-	-	-	31.61	115538.96
6	*Ceratoscopelusmaderensis* (Lowe, 1839)	Chordata	25.74	17.16	-	-	-	-
7	*Chauliodussloani* Bloch & Schneider, 1801	Chordata	8.58	514.71	9.04	90.43	-	-
8	*Chimaeramonstrosa* Linnaeus, 1758	Chordata	77.21	11323.57	63.3	15192.18	126.44	80608.58
9	*Coelorinchuscaelorhincus* (Risso, 1810)	Chordata	1466.92	48039.39	135.64	6330.08	71.13	4109.46
10	*Congerconger* (Linnaeus, 1758)	Chordata	-	-	-	-	31.61	65751.31
11	*Dalatiaslicha* (Bonnaterre, 1788)	Chordata	-	-	9.04	48832.01	-	-
12	*Diaphusholti* Tåning, 1918	Chordata	-	-	9.04	63.3	-	-
13	*Diaphusmetopoclampus* (Cocco, 1829)	Chordata	25.74	257.35	-	-	-	-
14	*Dipturusoxyrinchus* (Linnaeus, 1758)	Chordata	8.58	11152	-	-	-	-
15	*Epigonusconstanciae* (Giglioli, 1880)	Chordata	-	-	9.04	90.43	-	-
16	*Etmopterusspinax* (Linnaeus, 1758)	Chordata	102.94	6356.64	153.73	8048.24	576.9	25352.19
17	*Galeusmelastomus* Rafinesque, 1810	Chordata	471.82	75945.13	244.16	64024.19	63.22	18453.04
18	*Helicolenusdactylopterus* (Delaroche, 1809)	Chordata	34.31	2830.89	36.17	7234.37	-	-
19	*Hoplostethusmediterraneus* Cuvier, 1829	Chordata	274.51	17156.93	271.29	33458.97	7.9	869.31
20	*Hygophumhygomii* (Lütken, 1892)	Chordata	-	-	18.09	36.17	-	-
21	*Hymenocephalusitalicus* Giglioli, 1884	Chordata	-	-	18.09	587.79	-	-
22	*Lampanyctuscrocodilus* (Risso, 1810)	Chordata	77.21	943.63	72.34	1446.87	276.6	4741.68
23	*Lampanyctuspusillus* (Johnson, 1890)	Chordata	-	-	-	-	23.71	79.03
24	*Lepidorhombusboscii* (Risso, 1810) *	Chordata	34.31	4718.15	-	-	-	-
25	*Lophiusbudegassa* Spinola, 1807 *	Chordata	8.58	8578.46	27.13	18085.93	7.9	17228.11
26	*Merlucciusmerluccius* (Linnaeus, 1758) *	Chordata	-	-	135.64	199578.24	31.61	24972.85
27	*Micromesistiuspoutassou* (Risso, 1827) *	Chordata	42.89	11323.57	-	-	31.61	9167.25
28	Myctophidae Gill, 1893	Chordata	42.89	42.89	-	-	-	-
29	*Nettastomamelanura* Rafinesque, 1810	Chordata	8.58	428.92	36.17	1989.45	-	-
30	*Nezumiasclerorhynchus* (Valenciennes, 1838)	Chordata	523.29	6948.55	922.38	14649.6	229.18	6006.13
31	*Notacanthusbonaparte* Risso, 1840	Chordata	-	-	9.04	180.86	55.32	790.28
32	*Pagellusbogaraveo* (Brünnich, 1768) *	Chordata	274.51	47181.55	63.3	24777.72	15.81	4899.74
33	*Phycisblennoides* (Brünnich, 1768) *	Chordata	437.5	14583.39	352.68	32554.67	260.79	47495.84
34	*Scyliorhinuscanicula* (Linnaeus, 1758) *	Chordata	8.58	2916.68	-	-	-	-
35	*Stomiasboa* (Risso, 1810)	Chordata	25.74	514.71	18.09	180.86	23.71	158.06
36	*Symphurusnigrescens* Rafinesque, 1810	Chordata	-	-	-	-	71.13	158.06
37	*Synchiropusphaeton* (Günther, 1861)	Chordata	8.58	171.57	-	-	-	-
38	*Trachyrincusscabrus* (Rafinesque, 1810)	Chordata	-	-	1510.18	348154.15	790.28	202311.73
39	*Triglalyra* Linnaeus, 1758	Chordata	8.58	343.14	-	-	-	-
40	*Anamathiarissoana* (Roux, 1828)	Arthropoda	34.31	85.78	-	-	-	-
41	*Bathynectesmaravigna* (Prestandrea, 1839)	Arthropoda	8.58	85.78	-	-	-	-
42	*Eusergestesarcticus* (Krøyer, 1855)	Arthropoda	25.74	42.89	-	-	118.54	94.83
43	*Polybiusdepurator* (Linnaeus, 1758)	Arthropoda	-	-	-	-	31.61	158.06
44	*Nephropsnorvegicus* (Linnaeus, 1758)*	Arthropoda	8.58	866.42	-	-	-	-
45	Paguridae Latreille, 1802	Arthropoda	-	-	99.47	542.58	7.9	197.57
46	*Parapenaeuslongirostris* (Lucas, 1846)	Arthropoda	8.58	42.89	9.04	54.26	23.71	316.11
47	*Pasiphaeamultidentata* Esmark, 1866	Arthropoda	25.74	17.16	18.09	72.34	79.03	150.15
48	*Pasiphaeasivado* (Risso, 1816)	Arthropoda	34.31	34.31	-	-	395.14	790.28
49	*Plesionikaacanthonotus* (Smith, 1882)	Arthropoda	8.58	25.74	-	-	-	-
50	*Plesionikaheterocarpus* (Costa, 1871)	Arthropoda	8.58	25.74	-	-	-	-
51	*Plesionikamartia* (Milne-Edwards, 1883)	Arthropoda	308.82	1887.26	253.2	1627.73	371.43	3003.06
52	*Polychelestyphlops* Heller, 1862	Arthropoda	368.87	154.41	868.12	2893.75	316.11	790.28
53	*Processa* spp.	Arthropoda	-	-	9.04	27.13	-	-
54	*Solenoceramembranacea* (Risso, 1816)	Arthropoda	17.16	34.31	-	-	-	-
55	*Abraliaveranyi* (Rüppell, 1844)	Mollusca	25.74	85.78	-	-	-	-
56	*Ancistroteuthislichtensteinii* [A. Férussac (in A. Férussac & d'Orbigny), 1835]	Mollusca	-	-	-	-	7.9	790.28
57	*Bathypolypussponsalis* (Fischer & Fischer, 1892)	Mollusca	25.74	857.85	-	-	-	-
58	*Heteroteuthisdispar* (Rüppell, 1844)	Mollusca	-	-	-	-	23.71	55.32
59	*Histioteuthisbonnellii* (A. Férussac, 1834)	Mollusca	-	-	9.04	3074.61	-	-
60	*Histioteuthisreversa* (Verrill, 1880)	Mollusca	-	-	27.13	1718.16	39.51	2212.78
61	*Illexcoindetii* (Vérany, 1839)*	Mollusca	171.57	22218.22	-	-	-	-
62	*Neorossiacaroli* (Joubin, 1902)	Mollusca	51.47	1715.69	9.04	90.43	-	-
63	*Todarodessagittatus* (Lamarck, 1798)*	Mollusca	102.94	59191.39	63.3	21703.12	23.71	15173.38
64	Gastropoda	Mollusca	-	-	0.11	3.32	8.86	0.13
65	*Actinaugerichardi* (Marion, 1906)	Cnidaria	-	-	-	-	2.53	0.13
66	*Funiculinaquadrangularis* (Pallas, 1766)	Cnidaria	0.35	3.5	-	-	-	-
67	*Isidellaelongata* (Esper, 1788)	Cnidaria	15.15-18.42	731.83	-	-	-	-
68	Other Cnidaria	Cnidaria	-	-	0.11	3.32	25.31	0.76
69	Echinoidea [*Gracilechinusacutus* (Lamarck, 1816), Cidaridae spp.]	Echinodermata	2.68	4.66	7.52	13.16	16.7	1.14
70	*Hymenodiscuscoronata* (Sars, 1871)	Echinodermata	-	-	1.11	14.38	-	-
71	Mäerl	Rhodophyta	-	-	0.11	0.33	-	-
	Total Biomass			402,495.11		857,434.17		652,544.41
