# Supplementary material for: Biodiversity monitoring in bamboo coral assemblages in the North Aegean Sea, eastern Mediterranean Basin
Source: Biodivers Data J. 2025 Aug 1;13:e135156. doi: 10.3897/BDJ.13.e135156 (PMC12334926; doi:10.3897/BDJ.13.e135156)
Supplement: Supplementary material 1 — List of species and number of individuals registered by sampling site [file bdj-13-e135156-s001.docx]

**Supplementary material 1:** List of species and number of individuals registered by sampling site. Site A: min 46 species, 772-800 specimens; Site B: min 38 species, 689 specimens; Site C: min 37 species, 552 specimens. * Species of commercial value.

|  | **Species** | **Phylum** | **Site A** | **Weight A (gr)** | **Site B** | **Weight B (gr)** | **Site C** | **Weight C (gr)** |
| --- | --- | --- | --- | --- | --- | --- | --- | --- |
| 1 | *Argyropelecus hemigymnus* (Cocco, 1829) | Chordata | 3 | 5 | - | - | - | - |
| 2 | *Bellottia apoda* (Giglioli, 1883) | Chordata | 1 | 2 | - | - | 1 | 3 |
| 3 | *Benthosema glaciale* (Reinhardt, 1837) | Chordata | - | - | 1 | 1 | 7 | 12 |
| 4 | *Centrolophus niger* (Gmelin, 1789) | Chordata | 2 | 4,900 | - | - | - | - |
| 5 | *Centrophorus uyato* (Rafinesque, 1810) | Chordata | - | - | - | - | 4 | 14,620 |
| 6 | *Ceratoscopelus maderensis* (Lowe, 1839) | Chordata | 3 | 2 | - | - | - | - |
| 7 | *Chauliodus sloani* (Bloch & Schneider, 1801) | Chordata | 1 | 60 | 1 | 10 | - | - |
| 8 | *Chimaera monstrosa* (Linnaeus, 1758) | Chordata | 9 | 1,320 | 7 | 1,680 | 16 | 10,200 |
| 9 | *Coelorinchus caelorhincus* (Risso, 1810) | Chordata | 171 | 5,600 | 15 | 700 | 9 | 520 |
| 10 | *Conger conger* (Linnaeus, 1758) | Chordata | - | - | - | - | 4 | 8,320 |
| 11 | *Dalatias licha* (Bonnaterre, 1788) | Chordata | - | - | 1 | 5400 | - | - |
| 12 | *Diaphus holti* (Tåning, 1918) | Chordata | - | - | 1 | 7 | - | - |
| 13 | *Diaphus metopoclampus* (Cocco, 1829) | Chordata | 3 | 30 | - | - | - | - |
| 14 | *Dipturus oxyrinchus* (Linnaeus, 1758) | Chordata | 1 | 1,300 | - | - | - | - |
| 15 | *Epigonus constanciae* (Giglioli, 1880) | Chordata | - | - | 1 | 10 | - | - |
| 16 | *Etmopterus spinax* (Linnaeus, 1758) | Chordata | 12 | 741 | 17 | 890 | 73 | 3,208 |
| 17 | *Galeus melastomus* (Linnaeus, 1758) | Chordata | 55 | 8,853 | 27 | 7,080 | 8 | 2,335 |
| 18 | *Helicolenus dactylopterus* (Delaroche, 1809) | Chordata | 4 | 330 | 4 | 800 | - | - |
| 19 | *Hoplostethus mediterraneus* (Cuvier, 1829) | Chordata | 32 | 2,000 | 30 | 3,700 | 1 | 110 |
| 20 | *Hygophum hygomii* (Lütken, 1892) | Chordata | - | - | 2 | 4 | - | - |
| 21 | *Hymenocephalus italicus* (Giglioli, 1884) | Chordata | - | - | 2 | 65 | - | - |
| 22 | *Lampanyctus crocodilus* (Risso, 1810) | Chordata | 9 | 110 | 8 | 160 | 35 | 600 |
| 23 | *Lampanyctus pusillus* (Johnson, 1890) | Chordata | - | - | - | - | 3 | 10 |
| 24 | *Lepidorhombus boscii* (Risso, 1810) * | Chordata | 4 | 550 | - | - | - | - |
| 25 | *Lophius budegassa* (Spinola, 1807) * | Chordata | 1 | 1,000 | 3 | 2,000 | 1 | 2,180 |
| 26 | *Merluccius merluccius* (Linnaeus, 1758) * | Chordata | - | - | 15 | 22,070 | 4 | 3,160 |
| 27 | *Micromesistius poutassou* (Risso, 1827) * | Chordata | 5 | 1,320 | - | - | 4 | 1,160 |
| 28 | Myctophidae (Gill, 1893) | Chordata | 5 | 5 | - | - | - | - |
| 29 | *Nettastoma melanura* (Rafinesque, 1810) | Chordata | 1 | 50 | 4 | 220 | - | - |
| 30 | *Nezumia sclerorhynchus* (Valenciennes, 1838) | Chordata | 61 | 810 | 102 | 1,620 | 29 | 760 |
| 31 | *Notacanthus bonaparte* (Risso, 1840) | Chordata | - | - | 1 | 20 | 7 | 100 |
| 32 | *Pagellus bogaraveo* (Brünnich, 1768) * | Chordata | 32 | 5,500 | 7 | 2,740 | 2 | 620 |
| 33 | *Phycis blennoides* (Brünnich, 1768) * | Chordata | 51 | 1,700 | 39 | 3,600 | 33 | 6,010 |
| 34 | *Scyliorhinus canicula* (Linnaeus, 1758) * | Chordata | 1 | 340 | - | - | - | - |
| 35 | *Stomias boa boa* (Risso, 1810) | Chordata | 3 | 60 | 2 | 20 | 3 | 20 |
| 36 | *Symphurus nigrescens* (Rafinesque, 1810) | Chordata | - | - | - | - | 9 | 20 |
| 37 | *Synchiropus phaeton* (Günther, 1861) | Chordata | 1 | 20 | - | - | - | - |
| 38 | *Trachyrincus scabrus* (Rafinesque, 1810) | Chordata | - | - | 167 | 38,500 | 100 | 25,600 |
| 39 | *Trigla lyra* (Linnaeus, 1758) | Chordata | 1 | 4,040 | - | - | - | - |
| 40 | *Anamathia rissoana* (Roux, 1828) | Arthropoda | 4 | 10 | - | - | - | - |
| 41 | *Bathynectes maravigna* (Prestandrea, 1839) | Arthropoda | 1 | 10 | - | - | - | - |
| 42 | *Eusergestes arcticus* (Krøyer, 1855) | Arthropoda | 3 | 5 | - | - | 15 | 12 |
| 43 | *Liocarcinus depurator* (Linnaeus, 1758) | Arthropoda | - | - | - | - | 4 | 20 |
| 44 | *Nephrops norvegicus* (Linnaeus, 1758) * | Arthropoda | 1 | 101 | - | - | - | - |
| 45 | Paguridae (Latreille, 1802) | Arthropoda | - | - | 11 | 60 | 1 | 25 |
| 46 | *Parapenaeus longirostris* (Lucas, 1846) | Arthropoda | 1 | 5 | 1 | 6 | 3 | 40 |
| 47 | *Pasiphaea multidentata* (Esmark, 1866) | Arthropoda | 3 | 2 | 2 | 8 | 10 | 19 |
| 48 | *Pasiphaea sivado* (Risso, 1816) | Arthropoda | 4 | 4 | - | - | 50 | 100 |
| 49 | *Plesionika acanthonotus* (Smith, 1882) | Arthropoda | 1 | 3 | - | - | - | - |
| 50 | *Plesionika heterocarpus* (Costa, 1871) | Arthropoda | 1 | 3 | - | - | - | - |
| 51 | *Plesionika martia* (Milne-Edwards, 1883) | Arthropoda | 36 | 220 | 28 | 180 | 47 | 380 |
| 52 | *Polycheles typhlops* (Heller, 1862) | Arthropoda | 43 | 180 | 96 | 320 | 40 | 100 |
| 53 | *Processa* spp. | Arthropoda | - | - | 1 | 3 | - | - |
| 54 | *Solenocera membranacea* (Risso, 1816) | Arthropoda | 2 | 4 | - | - | - | - |
| 55 | *Abralia veranyi* (Rüppell, 1844) | Mollusca | 3 | 10 | - | - | - | - |
| 56 | *Ancistroteuthis lichtensteinii* (Férussac, 1835) | Mollusca | - | - | - | - | 1 | 100 |
| 57 | *Bathypolypus sponsalis* (Fischer & Fischer, 1892) | Mollusca | 3 | 100 | - | - | - | - |
| 58 | *Heteroteuthis dispar* (Rüppell, 1844) | Mollusca | - | - | - | - | 3 | 7 |
| 59 | *Histioteuthis bonnellii* (Férussac, 1834) | Mollusca | - | - | 1 | 340 | - | - |
| 60 | *Histioteuthis reversa* (Verrill, 1880) | Mollusca | - | - | 3 | 190 | 5 | 280 |
| 61 | *Illex coindetii* (Vérany, 1839) * | Mollusca | 20 | 2,590 | - | - | - | - |
| 62 | *Neorossia caroli* (Joubin, 1902) | Mollusca | 6 | 200 | 1 | 10 | - | - |
| 63 | *Todarodes sagittatus* (Lamarck, 1798) * | Mollusca | 12 | 6,900 | 7 | 2,400 | 3 | 1,920 |
| 64 | Gastropoda (Cuvier, 1795) | Mollusca | - | - | 1 | 30 | 1 | 70 |
| 65 | *Actinauge richardi* (Marion, 1906) | Cnidaria | - | - | - | - | 1 | 20 |
| 66 | *Funiculina quadrangularis* (Pallas, 1766) | Cnidaria | 3 | 30 | - | - | - | - |
| 67 | *Isidella elongata* (Esper, 1788) | Cnidaria | 130-158 | 6278 | - | - | - | - |
| 68 | Other Cnidaria | Cnidaria | - | - | 1 | 30 | 6 | 200 |
| 69 | Echinoidea (*Gracilechinus acutus*, Cidaridae spp.) | Echinodermata | 23 | 40 | 68 | 119 | 9 | 132 |
| 70 | *Hymenodiscus coronata* (Sars, 1871) | Echinodermata | - | - | 10 | 130 | - | - |
| 71 | Mäerl | Rhodophyta | - | - | 1 | 3 | - | - |
|  | **Total** |  | 772-800 | 57,343 | 689 | 95,126 | 552 | 82,993 |
