# Supplementary material for: Biodiversity monitoring in bamboo coral assemblages in the North Aegean Sea, eastern Mediterranean Basin
Source: Biodivers Data J. 2025 Aug 1;13:e135156. doi: 10.3897/BDJ.13.e135156 (PMC12334926; doi:10.3897/BDJ.13.e135156)
Supplement: Supplementary material 2 — BLAST results [file bdj-13-e135156-s002.docx]

**Supplementary material 2:** Details and Blast results from all specimens used in this study.

| **A/A** | **Sample ID** | **Gene** | **Top BLAST matches** | **%** | **Accession Number** |
| --- | --- | --- | --- | --- | --- |
| 1 | IS1 | *igr4* | *Acanella* sp., *Acanella eburnea*, *Acanella arbuscula*, *Orstomisis crosniery* | 100, 100, 100, 99.73 | PV285286 |
| 2 | IS2 | *igr4* | *Acanella* sp., *Acanella eburnea*, *Acanella arbuscula*, *Orstomisis crosniery* | 100, 100, 100, 99.73 | PV285287 |
| 3 | IS3 | *igr4* | *Acanella* sp., *Acanella eburnea*, *Acanella arbuscula*, *Orstomisis crosniery* | 100, 100, 100, 99.73 | PV285288 |
| 4 | IS4 | *igr4* | *Acanella* sp., *Acanella eburnea*, *Acanella arbuscula* | 100, 100, 100 | PV285289 |
| 5 | IS5 | *igr4* | *Acanella* sp., *Acanella eburnea*, *Acanella arbuscula* | 100, 100, 100 | PV285290 |
| 6 | IS6 | *igr4* | *Acanella* sp., *Acanella eburnea*, *Acanella arbuscula, Plimarella adhaerans* | 100, 100, 100, 99.28 | PV285291 |
| 7 | IS5 | mtMutS | *Acanella* sp., *Acanella eburnea*, *Acanella cf. eburnea*, *Isidella elongata* | 100, 99.89, 100, 98.52 | PV285292 |
