# Supplementary material for: Biodiversity monitoring in bamboo coral assemblages in the North Aegean Sea, eastern Mediterranean Basin
Source: Biodivers Data J. 2025 Aug 1;13:e135156. doi: 10.3897/BDJ.13.e135156 (PMC12334926; doi:10.3897/BDJ.13.e135156)
Supplement: Supplementary material 3 — Analysis of similarity percentage (SIMPER) [file bdj-13-e135156-s003.docx]

**Supplementary material 3:** Analysis of similarity percentage (SIMPER) for assemblages among Sites A, B, and C, and of dissimilarity percentage among hauls. Contrib. %: contribution in %; Cumulative %: cumulative contribution in %. Species are listed in decreasing order of AD among hauls.

| **Species** | **Phylum** | **Contrib. %** | **Cumulative %** |
| --- | --- | --- | --- |
| *Trachyrincus scabrus* | Chordata | 16.24 | 16.24 |
| *Nezumia sclerorhynchus* | Chordata | 11.08 | 27.32 |
| *Coelorinchus caelorhincus* | Chordata | 10.85 | 38.17 |
| *Polycheles typhlops* | Arthropoda | 10.70 | 48.87 |
| *Phycis blennoides* | Chordata | 7.253 | 56.12 |
| *Plesionika martia* | Arthropoda | 6.609 | 62.73 |
| *Etmopterus spinax* | Chordata | 6.606 | 69.34 |
| *Galeus melastomus* | Chordata | 5.088 | 74.42 |
| *Hoplostethus mediterra* | Chordata | 3.515 | 77.94 |
| *Lampanyctus crocodilus* | Chordata | 3.337 | 81.28 |
| *Pasiphaea sivado* | Arthropoda | 3.069 | 84.35 |
| *Pagellus bogaraveo* | Chordata | 2.288 | 86.63 |
| *Chimaera monstrosa* | Chordata | 1.982 | 88.62 |
| *Merluccius merluccius* | Chordata | 1.118 | 89.73 |

| **Species** | **Phylum** | **Contrib. %** | **Cumulative %** |
| --- | --- | --- | --- |
| *Polycheles typhlops* | Arthropoda | 16.49 | 16.49 |
| *Trachyrincus scabrous* | Chordata | 12.25 | 28.74 |
| *Plesionika martia* | Arthropoda | 10.18 | 38.91 |
| *Nezumia sclerorhynchus* | Chordata | 8.288 | 47.20 |
| *Coelorinchus caelorhincus* | Chordata | 8.076 | 55.28 |
| *Phycis blennoides* | Chordata | 5.446 | 60.72 |
| *Etmopterus spinax* | Chordata | 5.029 | 65.75 |
| *Pasiphaea sivado* | Arthropoda | 4.291 | 70.04 |
| *Galeus melastomus* | Chordata | 3.795 | 73.84 |
| *Hoplostethus mediterraneus* | Chordata | 2.616 | 76.45 |
| *Lampanyctus crocodilus* | Chordata | 2.537 | 78.99 |
| *Todarodes sagittatus* | Mollusca | 2.082 | 81.07 |
| *Illex coindetii* | Mollusca | 1.743 | 82.82 |
| *Pagellus bogaraveo* | Chordata | 1.704 | 84.52 |
| *Chimaera monstrosa* | Chordata | 1.499 | 86.02 |
| *Eusergestes arcticus* | Arthropoda | 1.491 | 87.51 |
| *Pasiphaea multidentata* | Arthropoda | 1.285 | 88.80 |
| Paguridae | Arthropoda | 1.057 | 89.85 |
